# Explainable graph neural network based on metabolic brain imaging for differential diagnosis of parkinsonism

**DOI:** 10.3389/fnagi.2025.1580910

**Published:** 2025-04-11

**Authors:** Ronghua Ling, Xingxing Cen, Shaoyou Wu, Min Wang, Ying Zhang, Juanjuan Jiang, Jiaying Lu, Yingqian Liu, Chuantao Zuo, Jiehui Jiang, Yinghui Yang, Zhuangzhi Yan

**Affiliations:** ^1^School of Communication and Information Engineering, Shanghai University, Shanghai, China; ^2^School of Medical Imaging, Shanghai University of Medicine and Health Science, Shanghai, China; ^3^Glorious Sun School of Business and Management, Donghua University, Shanghai, China; ^4^School of Life Sciences, Shanghai University, Shanghai, China; ^5^Department of Nuclear Medicine and PET Center, National Clinical Research Center for Aging and Medicine, National Center for Neurological Disorders, Huashan Hospital, Fudan University, Shanghai, China; ^6^School of Electrical Engineering, Shandong University of Aeronautics, Binzhou, China; ^7^College of Health Management, Shanghai Jian Qiao University, Shanghai, China

**Keywords:** parkinsonism, glucose metabolism, graph neural network, brain imaging, classification

## Abstract

**Background:**

Accurate differentiation of parkinsonian syndromes remains challenging due to overlapping clinical manifestations and subtle neuroimaging variations. This study introduces an explainable graph neural network (GNN) framework integrating a Regional Radiomics Similarity Network (R2SN) and Transformer-based attention mechanisms to address this diagnostic dilemma.

**Methods:**

Our study prospectively enrolled 1,495 participants, including 220 healthy controls and 1,275 patients diagnosed with idiopathic Parkinson’s disease (IPD), multiple system atrophy (MSA), or progressive supranuclear palsy (PSP), all undergoing standardized ^18^F-fluorodeoxyglucose positron emission tomography imaging. Metabolic networks were constructed by encoding edge weights derived from radiomic feature similarity matrices, enabling simultaneous quantification of microscopic metabolic heterogeneity and macroscale network reorganization.

**Results:**

The proposed framework achieved superior classification performance with F1-scores of 92.5% (MSA), 96.3% (IPD), and 86.7% (PSP), significantly outperforming comparators by 5.5–8.3%. Multiscale interpretability analysis revealed: (1) Regional hypometabolism in pathognomonic nodes (putamen in IPD, midbrain tegmentum in PSP); (2) Disease-specific connectivity disruptions (midbrain-prefrontal disconnection in PSP, cerebellar-pontine decoupling in MSA). The substructure attention mechanism reduced computational complexity by 41% while enhancing diagnostic specificity (PSP precision +5.2%).

**Conclusion:**

The proposed R2SN-based explainable GNN framework for parkinsonian syndrome differentiation establishes a new paradigm for precision subtyping of neurodegenerative disorders, with methodological extensibility to other network-driven neurological conditions.

## 1 Introduction

Atypical parkinsonian syndromes (PDS) are a group of progressive neurodegenerative disorders that impair the nervous system and motor functions controlled by neural pathways. These syndromes present with parkinsonism, marked by bradykinesia, rigidity, and postural instability, yet they differ from idiopathic Parkinson’s disease (IPD) in clinical manifestations, progression, and underlying pathology (Vidailhet, 2011). The clinical differential diagnosis between atypical PDS can be challenging and critical due to their potential to lead to vastly different PDS. The clinical phenotype of IPD is primarily characterized by asymmetric parkinsonism, which is responsive to levodopa (Nido et al., 2024). This phenotype closely resembles the clinical features of multiple system atrophy (MSA) and progressive supranuclear palsy (PSP) (Wu et al., 2022). Previous studies have demonstrated that approximately 20 to 30% of patients initially diagnosed with IPD were later found to have MSA or PSP upon pathological examination (Hughes et al., 2002). Accurate differentiation between these syndromes is essential, as it significantly impacts clinical management and prognosis. A thorough understanding of the pathological characteristics, incidence, and clinical implications of PDS is vital for both clinicians and neuroscientist.

Neuroimaging, particularly positron emission tomography (PET) with ^18^F-fluorodeoxyglucose (FDG) tracing, is widely employed to identify a range of glucose metabolism abnormalities in neurodegenerative diseases (Kreisl et al., 2020). Distinct metabolic patterns are observed among IPD, MSA, and PSP (Wu et al., 2022; Zhao et al., 2022). Compared to similar magnetic resonance imaging (MRI) techniques, PET has garnered significant clinical attention due to its superior sensitivity in detecting metabolic changes. In IPD, PET typically reveals increased glucose metabolism in the striatum, thalamus, motor cortex, and cerebellum, with potential decreases observed in the temporoparietooccipital cortex (Wang et al., 2024; Meyer et al., 2017; Saeed et al., 2020). In contrast, MSA is characterized by decreased glucose metabolism in the posterior putamen and cerebellum, distinguishing it from IPD (Kim et al., 2021; Xue et al., 2024). PSP, on the other hand, shows reduced glucose metabolism in the medial and dorsolateral frontal cortex, caudate, thalamus, and upper brainstem (Isella et al., 2022). Various approaches based on visual evaluation and metabolic pattern analysis have been proposed to identify these metabolic abnormalities. These methods are instrumental in developing more effective tools for the differential PDS diagnosis.

These statistical methods primarily emphasize relative metabolic alterations, such as hypermetabolism or hypometabolism, but struggle to characterize subtle metabolic distinctions between disease subtypes. Recent advancements have employed radiomics to address this limitation (Sun et al., 2024; Hu et al., 2021; Wu et al., 2013). By extracting multi-level, high-dimensional information from metabolically aberrant regions, radiomics enhances the granularity of metabolic assessments. However, a key limitation of this approach is its reliance on manually defined features, which are often mathematically or semantically derived from expert knowledge. To overcome this, recent studies have proposed integrating deep learning techniques to extract more complex, higher-dimensional features from PET images (Cho et al., 2021; Ling et al., 2024). These data-driven methods are then combined with radiomics frameworks to improve interpretability, creating a hybrid strategy that merges the superior classification capabilities of deep learning with the explanatory power of traditional radiomics (Ling et al., 2024; Jiang et al., 2024). While the integration with radiomics adds a layer of interpretability, the transparency of these hybrid features still requires further refinement to enhance their clinical applicability and ensure robust interpretability in real-world settings.

Graph Neural Networks (GNNs) have emerged as a powerful tool in deep learning, particularly for applications involving structured data, such as graphs (Zheng et al., 2024). By treating brain regions as nodes and their interactions as edges, GNNs can capture both functional and structural connectivity patterns, providing valuable insights into the brain’s organization (Cui et al., 2023). However, the design of GNNs inherently limits their ability to model global relationships due to their small receptive fields and reliance on localized feature processing. To address this, Transformer models were introduced, leveraging larger receptive fields to capture long-range dependencies and global information (Sun et al., 2025). Additionally, recent advancements in neuroscience have introduced a novel network approach known as the Regional Radiomics Similarity Network (R2SN) (Zhao et al., 2024; Zhao et al., 2021). This macroscale network, based on the similarity of radiomic features, offers robustness, stability, and a biological basis. R2SN can reflect subtle changes in brain structure and metabolism, providing a deeper understanding of the human brain through structural and metabolic imaging (Zhao et al., 2023). The R2SN offers substantial promise for elucidating cognitive brain mechanisms and discovering hierarchical structural impairments linked to neurodegenerative disorders. The integration of GNNs with R2SN represents an emerging research direction in graph representation learning, particularly for the differential diagnosis of PDS. By combining these two models, we can extend the receptive field, capture more intricate graph structures, and enhance the information transfer mechanism, thereby improving the interpretability of deep learning models. This integration also streamlines the model architecture, reduces computational costs, and enhances training efficiency, making it a promising approach for clinical neuroimaging applications. Shapley additive explanations (SHAP) and Local interpretable model-agnostic explanations (LIME) are widely used for explaining non-graph data; however, they struggle to capture interaction effects specific to graph structures. GNNExplainer addresses this limitation by optimizing the importance of subgraph structures and node features, thereby providing interpretable explanations for graph-based predictions.

The primary objective of this study is to introduce a novel, explainable GNN attention framework based on FDG PET imaging for the differential diagnosis of PDS. First, we constructed a glucose metabolic representation graph, where nodes represented brain regions and network connectivity was defined by the radiomic feature similarity between pairs of regions. The proposed GNN model then employed a transformer-based graph attention network to process this metabolic representation graph and predict the differential diagnosis of PDS. Finally, we utilized GNNExplainer, an interpretable model-agnostic tool, to identify the key factors contributing to the differential diagnosis of PDS and to detect abnormal metabolic regions characterized by representative features (Li et al., 2024; Ying et al., 2019). The principal contributions of this study are as follows: (1) We propose a novel, explainable GNN-Transformer attention framework for the diagnosis of PDS. By synergistically integrating graph structural priors and global attention mechanisms, this approach enhances neural representational capacity to model long-range node dependencies and hierarchical subgraph patterns, significantly improving discrimination accuracy between IPD and atypical parkinsonian syndromes (e.g., PSP, MSA). (2) Local attention mechanisms are applied to substructure tags and their corresponding nodes, with masks employed to restrict the focus of substructure tags to the relevant nodes. (3) We employed GNNExplainer, a model-agnostic tool, to interpret key factors in the predictions made by the graph neural network and to detect abnormal metabolic modules associated with PDS, providing a deeper understanding of the disease’s underlying mechanisms.

## 2 Materials and methods

### 2.1 Participants

**Figure 1:**
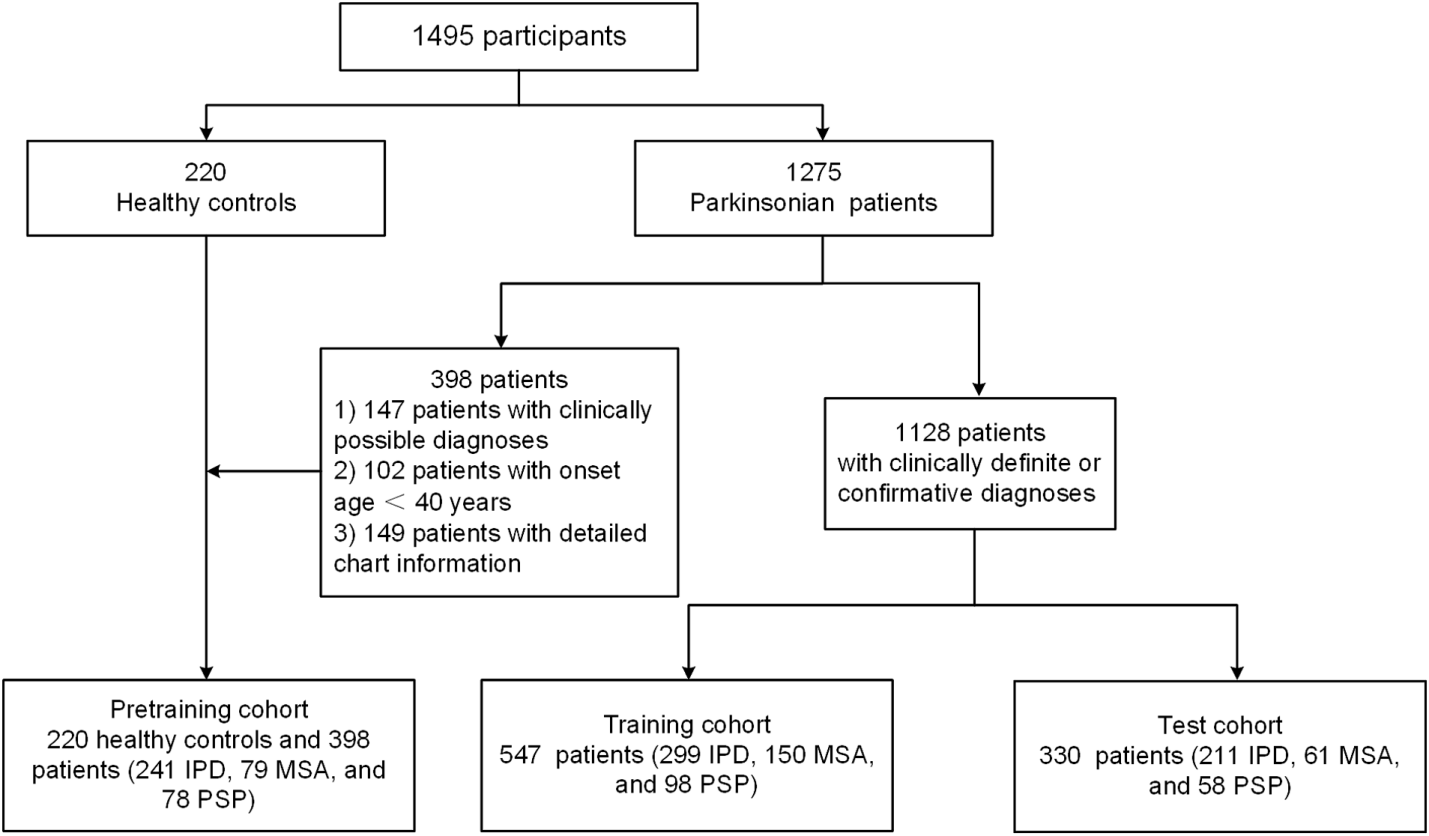
The flowchart of all participants in this study.

A total of 1,495 participants underwent FDG PET scanning, comprising 220 healthy controls and 1,275 patients diagnosed with IPD, MSA, and PSP. These individuals were enrolled from the Huashan parkinsonian PET Imaging (HPPI) Database, established by the Department of Nuclear Medicine and PET Center at Huashan Hospital, Fudan University. All procedures involving human participants adhered to the ethical standards set by the institutional and national research committees, as well as the 1964 Declaration of Helsinki and its subsequent amendments. The data analysis and ethical approval for this study were granted by the institutional review board of Huashan Hospital (identifier: KY2011-174, Date: 24 August 2011), and informed consent was obtained from all participants. A detailed eligibility profile of these subjects is shown in Figure 1.

We investigated utilizing three distinct cohorts to develop and evaluate our proposed explainable GNN attention model, as previously described (Wu et al., 2022; Ling et al., 2024). The pre-training cohort comprised 241 patients with IPD, 79 with MSA, and 78 with PSP, all presenting with clinically possible diagnoses. Additionally, individuals with an onset age younger than 40 years and those with definitive clinical diagnoses lacking detailed medical records were included in this cohort. This group was utilized for the preliminary training of the parkinsonian differential diagnosis network. The training cohort consisted of 299 IPD patients, 150 MSA patients, and 98 PSP patients, all with clinically definite diagnoses. This cohort was employed to fine-tune the initial DL model. The test cohort included 211 IPD patients, 61 MSA patients, and 58 PSP patients, all with clinically confirmed diagnoses. This group was used to assess the predictive performance of the model.

### 2.2 PET acquisition and preprocessing

After attenuation correction performed using low-dose CT, the emission scan was acquired at 60-min post injection of approximately 185 MBq ^18^F-FDG and lasted 10 min (Siemens Biograph 64 HD PET/CT, Siemens, Germany). PET images were reconstructed by using the ordered subset expectation maximization method following corrections for scatter, dead time, and random coincidence. High-resolution T1-weighted MRI images were acquired using the following parameters (Discovery MR750; GE Medical Systems): echo time (TE), 3.2 ms; repetition time (TR), 8.2 ms; inversion time (TI), 450 ms; flip angle, 12^o^; 25.6 cm field of view (FOV); acquisition matrix, 256 × 256 × 152; and voxel size, 1 × 1 × 1 mm. The obtained FDG PET images were realigned, averaged, and spatially coregistered with their corresponding MRI images using SPM12 software (Wellcome Department of Imaging Neuroscience, Institute of Neurology, London, UK) implemented in MATLAB 2021b (MathWorks Inc., Sherborn, MA). Then, the FDG PET images were normalized to the Montreal Neurological Institute standard space via applying the MRI-segmented parameters. Subsequently, the normalized PET images were smoothed with a three-dimensional Gaussian filter with a full width at half maximum (FWHM) of 10 mm. Local glucose metabolic activity, normalized to global activity, was quantified using the standardized uptake value ratio (SUVR).

### 2.3 R2SN based metabolic network

All FDG-PET images were utilized to construct independent metabolic network models using the R2SN method, which incorporates nodes, edges, and node features (Zhao et al., 2024). Initially, 80 cortical and 16 subcortical regions of interest (ROIs) were identified using automated anatomical labeling (AAL) (Tzourio-Mazoyer et al., 2002) in conjunction with the PD25 atlas (Xiao et al., 2015). A total of 107 radiomic features were extracted with the open-source software PyRadiomics, capturing information on shape, glucose uptake distribution, texture (including gray-level co-occurrence matrix [GLCM], gray-level run-length matrix [GLRLM], and gray-level size zone matrix [GLSZM]), and margin characteristics (neighborhood gray-tone difference matrix [NGTDM]) for each brain region. Features exhibiting high collinearity (*r* > 0.9, Pearson correlation) were deemed redundant, resulting in a final set of 36 radiomic features for subsequent analysis. Next, the standard min-max normalization method was applied to harmonize radiomic features across different brain regions for each subject. Individual R2SNs were then generated by mapping each subject’s normalized radiomic features into a pairwise inter-regional Pearson correlation matrix. Consequently, a 96 × 96 metabolic network matrix was produced for each subject, where nodes correspond to the defined brain regions and edges are determined by the Pearson correlation coefficients between the radiomic features of these regions.

### 2.4 Explainable GNN attention model

**Figure 2:**
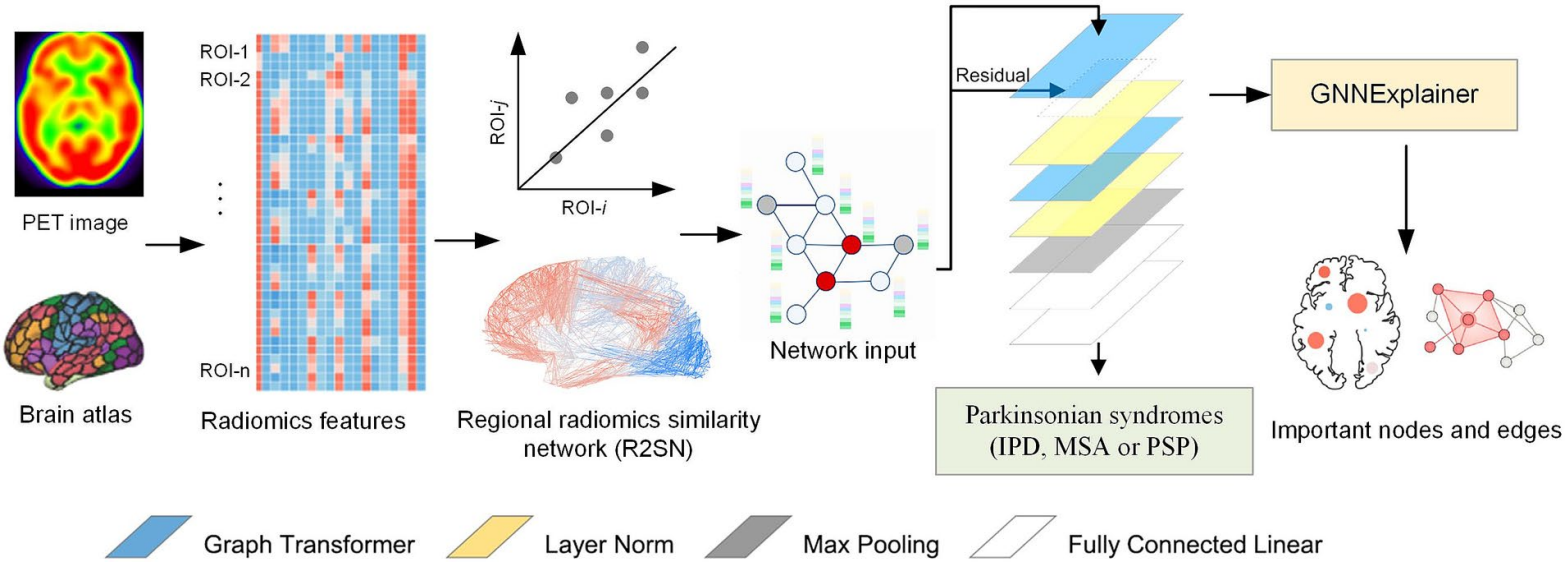
Overview of the propose explainable Graph Neural Network model.

This section introduces the structure of the proposed model and its detailed information. As shown in Figure 2, the proposed multimodal graph neural network processes brain connectivity graphs through a hierarchical architecture comprising four principal components: (1) graph transformer layers with hybrid attention mechanisms, (2) Classifier Module, and (3) explainability module. As illustrated in Figure 1, the model operates on weighted graphs $G=\left(V,E,X,W\right)$ , where nodes $v_{i}\epsilon \;V$ represent 96 defined ROIs with 36-dimensional selected radiomic feature vectors $x_{i}\in {\mathbb{R}}^{36}$ encoding metabolic measurements, and edges $e_{ij}\in E$ are weighted by standard transformed Pearson correlation coefficients $w_{ij}\in \left[0,1\right]$ computed from FDG PET signals.

Graph transformer module employs three stacked layers combining global and localized attention mechanisms. Each layer implements: multi-head graph attention (4 heads) computing cross-node interactions through scaled dot-product attention:


$$\mathrm{Attention}\left(Q,K,V\right)=\mathrm{softmax}\left(\frac{QK^{T}}{\sqrt{d_{k}}}⊙W\right)V$$

where $Q=XW_{Q},K=XW_{K}\text{,}$ and $V={XW}_{V}$ with learnable projections $W_{Q},W_{K}\in {\mathbb{R}}^{36\times 24},W_{V}\in {\mathbb{R}}^{36\times 32}$ , The Hadamard product ⊙W preserves original connectivity strengths.

Learnable structure tokens $s\in {\mathbb{R}}^{36}$ interact with local features via gated fusion:


$$g_{i}=\sigma \left(W_{g}\left[x_{i}\|s\right]\right),\tilde{x_{i}}=g_{i}⊙x_{i}+\left(1-g_{i}\right)⊙s$$

with $W_{g}\in {\mathbb{R}}^{72\times 36}$ controlling cross-scale information flow. Position-wise feedforward network applies nonlinear transformation:


$$FFN\left(x\right)=W_{2}\left(\mathrm{GELU}\left(W_{1}x+b_{1}\right)\right)+b_{2}$$

where $W_{1}\in {\mathbb{R}}^{144\times 36}$ , $W_{2}\in {\mathbb{R}}^{36\times 144}$ . Residual connections and layer normalization yield final outputs:


$$X^{\left(l+1\right)}=\mathrm{LayerNorm}\left(X^{l}+\mathrm{Attention}\left(X^{l}\right)+FFN\left(X^{l}\right)\right)$$

Interpretable classification head aggregates multi-scale graph representations through:


$$h_{\mathrm{pool}}=\max_{i\in \left(1,\ldots ,96\right)}\left(X_{i}^{3}\right),h_{\mathrm{concat}}=\left[h_{\mathrm{pool}}^{\left(1\right)}\|h_{\mathrm{pool}}^{\left(2\right)}\|h_{\mathrm{pool}}^{\left(3\right)}\right]$$

where ∥ denotes concatenation. The classifier implements:


$$p\left(y|c\right)=\mathrm{softmax}\left(W_{c}\left(\mathrm{GELU}\left(W_{b}\left(\mathrm{GELU}\left(W_{a}h_{\mathrm{concat}}\right)\right)\right)\right)\right)$$

with $W_{a}\in {\mathbb{R}}^{128\times 108}$ , $W_{b}\in {\mathbb{R}}^{64\times 128}$ , and $W_{c}\in {\mathbb{R}}^{3\times 64}$ . Dropout (*p* = 0.3) and LayerNorm operations precede each lineartransformation.

An enhanced GNNExplainer methodology was employed to interpret the contribution of brain regions (nodes) and metabolic interactions (edges) for glucose activity (Zhao et al., 2024). The proposed explainable GNN framework incorporates an enhanced GNNExplainer module to elucidate disease-specific biomarkers and connectivity patterns underlying the model’s diagnostic decisions. This interpretability analysis operates through the following synergistic components: Node-wise importance scores are computed via gradient-based attribution, measuring the sensitivity of classification probability to input feature perturbations:


$$I_{\mathrm{node}}\left(i\right)=∥\frac{\partial \mathrm{logp}\left(y|G\right)}{\partial x_{i}}{∥}_{2}\in {\mathbb{R}}^{+}$$

where $x_{i}\in {\mathbb{R}}^{36}$ denotes the feature vector of node $v_{i}$ . Edge importance is derived by aggregating attention weights across all transformer layers, retaining top 15% connections:


$$\begin{array}{l} I_{\mathrm{edge}}\left(i,j\right)=\frac{1}{L}\sum_{l=1}^{3}A_{i,j}^{\left(l\right)}\;\mathrm{with}\;A_{i,j}^{\left(l\right)}=\\ \mathrm{softmax}\left(\frac{\left({\left(W_{Q}x_{i}^{\left(l\right)}\right)}^{T}\left(W_{k}x_{j}^{\left(l\right)}\right)\right)}{\sqrt{d_{k}}}\right) \end{array}$$

Critical nodes are mapped to neuroanatomical structures using the defined ROIs, revealing consistent involvement of basal ganglia-thalamocortical circuits. Disease-specific connectivity alterations are quantified through Jaccard similarity analysis.

The model is optimized through a carefully designed training protocol that balances convergence speed with generalization performance. The objective function combines cross-entropy loss with regularization terms:


$$L=-\sum_{c=1}^{3}y_{c}\log p_{c}+10^{-4}\|\theta {\|}_{2}^{2}+0.2L_{\mathrm{SupCon}}$$

where the supervised contrastive term ${ℒ}_{\mathrm{SupCon}}=\sum_{i\epsilon ℬ}\frac{1}{|\left(i\right)|}\sum_{p\in \left(i\right)}\log\frac{\exp\left(z_{i}z_{p}/0.07\right)}{\sum_{a\in A\left(i\right)}\exp\left(z_{i}z_{p}/0.07\right)}$ enhances feature discriminability through normalized temperature-scaled cosine similarity in latent space. Optimization is performed using the Adam algorithm with $\beta_{1}=0.9,\beta_{2}=0.999$ , and $\in =10^{-8}\text{,}$ where the learning rate follows a phased schedule: initial linear warmup from 0 to $5\times 10^{-4}$ over 5 epochs, followed by cosine decay to $10^{-6}$ cross the remaining training span. To ensure numerical stability, global gradient norms are clipped at 3.0 using the adaptive clipping algorithm, while mixed-precision training with dynamic loss scaling (FP16 arithmetic) accelerates computation without sacrificing precision. Regularization is implemented through a multi-tiered strategy: (1) spatial dropout randomly masks 15% of node features at transformer inputs, (2) attention dropout with probability 0.1 is applied to attention weights, (3) feature dropout (*p* = 0.4) in fully connected layers, and (4) stochastic edge perturbation where 10% of connections receive Gaussian noise. The model trains for maximum 200 epochs with batch size 32, employing stratified sampling to maintain class balance. An early stopping monitor tracks validation loss with patience of 10 epochs, reverting to the best-performing weights upon termination. Computational implementation leverages 4 NVIDIA 4090 GPUs through data parallelism.

### 2.5 Methods comparison

The proposed explainable GNN framework was benchmarked against conventional and deep learning approaches to evaluate its efficacy for the differential diagnosis of PDS. SUVR analysis served as the clinical reference, quantifying regional tracer uptake in predefined dopaminergic pathways normalized to cerebellar reference values. Radiomics pipelines extracted handcrafted PET features followed by feature selection and support vector machines (SVM) classification, representing classical machine learning paradigms. For deep learning comparisons, ResNet-50, ResNet-101 and DenseNet-121 were adapted by reshaping functional connectivity matrices into pseudo-images, imposing grid-based convolutional operations on the non-Euclidean brain network data. Graph-based baselines included Graph convolutional network (GCN), BrainNetCNN (specialized edge-to-edge filters), and Transformer-GCN hybrids (self-attention augmented message passing). The proposed explainable GNN advances these methods through 2 key innovations: (1) multi-scale attention fusion combining global interactions and localized substructure encoding; (2) anatomically grounded interpretability linking saliency maps to neurobiological substrates. Unlike SUVR’s reliance on predefined regions or CNNs’ grid biases, the GNN intrinsically models brain network topology, while outperforming prior GNNs through adaptive sparsification and hierarchical feature learning. This methodology comparison underscores the necessity of domain-specific architectural design for neurodegenerative disorder analysis.

## 3 Results

### 3.1 Demographic information

Table 1 summarizes the clinical and demographic characteristics of the study cohort. Notably, patients diagnosed with PSP exhibited a significantly higher mean age compared to other diagnostic groups (*p* < 0.001). Intergroup analysis revealed marked disparities in neuropsychological assessments across PDS subtypes (*p* < 0.001), particularly evident in IPD cases demonstrating comparatively reduced disease severity as measured by Hoehn and Yahr staging and UPDRS evaluations. Gender distribution remained comparable among all cohorts, with no statistically significant variations observed (*p* > 0.05 for all comparisons).

**Table 1:** Clinical and demographic characteristics of all patients.

Cohort	Characteristic	IPD	MSA	PSP	*p* value
Pretrain cohort	*n*	241	79	78	/
	Sex (male /female)	154/87	42/37	45/33	0.202
	Age (years)	50.0 ± 15.2	57.5 ± 10.6	64.6 ± 8.6	<0.001
	Symptom duration (months)	/	/	/	/
	Hoehn and Yahr stage	/	/	/	/
	UPDRS	/	/		/
Train cohort	*N*	299	150	98	/
	Sex (male/female)	166/133	78/72	60/38	0.359
	Age (years)	60.2 ± 8.5	57.8 ± 8.0	67.2 ± 8.0	<0.001
	Symptom duration (months)	45.3 ± 46.0	24.3 ± 17.1	35.0 ± 20.7	<0.001
	Hoehn and Yahr stage	2.2 ± 1.0	3.1 ± 0.8	3.2 ± 0.8	<0.001
	UPDRS	27.0 ± 14.3	30.6 ± 14.5	30.1 ± 13.5	0.02
Test cohort	*n*	211	61	58	/
	Sex (male/female)	130/81	32/29	39/19	0.241
	Age (years)	60.0 ± 7.6	58.5 ± 6.3	65.1 ± 6.6	<0.001
	Symptom duration (months)	39.0 ± 41.3	27.0 ± 20.1	34.1 ± 22.7	0.062
	Hoehn and Yahr stage	1.9 ± 0.9	2.9 ± 0.8	3.0 ± 0.8	<0.001
	UPDRS	22.8 ± 12.1	29.3 ± 14.4	26.8 ± 11.0	<0.001

UPDRS, Unified Parkinson’s Disease Rating Scale; IPD, idiopathic Parkinson’s disease; MSA, multiple system atrophy; PSP, progressive supranuclear palsy.

### 3.2 Classification results

The proposed explainable GNN model demonstrated superior diagnostic capabilities across PDS subtypes compared to benchmark methods, as detailed in Table 2. For MSA, our proposed explainable GNN model achieved state-of-the-art performance with recall = 91.8% ± 1.6, precision = 93.4% ± 1.2, and F1-score = 92.5% ± 1.5, surpassing all comparative models by 5.5–8.3% in F1-score (*p* < 0.01, paired t-test). This substantial improvement highlights the model’s enhanced capacity to capture MSA-specific pathophysiological patterns through its substructure-aware attention mechanisms. In IPD classification, our proposed explainable GNN mode attained optimal recall (96.1% ± 0.4) and F1-score (96.3% ± 0.3), though precision (96.5% ± 0.7) showed a marginal 0.1% deficit compared to ResNet-101 (96.5% ± 0.6). This discrepancy arises from the model’s conservative thresholding strategy to minimize false negatives—a clinically preferable tradeoff given IPD’s progressive nature requiring early intervention. For PSP, the framework achieved peak precision (85.4% ± 2.6) and F1-score (86.7% ± 0.9), with recall (88.3% ± 2.1) slightly lower than ResNet-101’s 88.6% ± 3.7. The higher F1-score (*p* = 0.013) confirms superior balance between specificity and sensitivity, particularly crucial for PSP’s differential diagnosis from atypical PDS.

**Table 2:** The classification comparison results of the proposed GNN model and other models for differential diagnosis of parkinsonian disorders in the test dataset.

Model	Multiple system atrophy			Idiopathic Parkinson’s disease			Progressive supranuclear palsy		
	Recall	Precision	F1-score	Recall	Precision	F1-score	Recall	Precision	F1-score
SVM	84.3 ± 2.5	90.3 ± 6.2	87.3 ± 3.4	92.2 ± 0.9	96.1 ± 0.6	94.1 ± 0.2	87.7 ± 5.4	72.4 ± 4.2	79.1 ± 1.7
Radiomics	85.1 ± 2.1	88.2 ± 4.6	86.6 ± 2.9	93.1 ± 0.8	95.6 ± 0.5	94.3 ± 0.4	85.2 ± 3.1	75.5 ± 3.2	80.0 ± 1.6
ResNet 50	87.6 ± 3.2	85.6 ± 3.2	86.5 ± 1.8	93.1 ± 0.6	94.5 ± 0.6	93.8 ± 0.6	85.5 ± 1.1	79.7 ± 6.2	82.4 ± 3.8
ResNet 101	87.4 ± 4.2	87.0 ± 4.0	87.2 ± 4.1	93.4 ± 0.6	**96.5 ± 0.6**	94.9 ± 0.7	**88.6 ± 3.7**	79.7 ± 3.5	83.8 ± 1.4
DenseNet-121	90.1 ± 4.1	92.5 ± 1.5	91.5 ± 2.9	94.3 ± 1.5	95.4 ± 0.8	94.9 ± 0.7	86.8 ± 2.1	81.8 ± 6.7	84.1 ± 2.9
GCN	90.0 ± 3.9	90.8 ± 3.2	90.4 ± 3.4	94.8 ± 1.1	94.9 ± 0.5	94.8 ± 0.3	84.2 ± 5.1	84.1 ± 6.3	83.7 ± 1.0
BrainNetCNN	87.9 ± 2.4	86.7 ± 2.3	87.1 ± 2.3	92.7 ± 3.8	95.6 ± 2.0	94.1 ± 2.2	85.1 ± 6.5	76.9 ± 11.1	80.2 ± 6.3
Transformer-GCN	90.6 ± 3.5	90.8 ± 5.9	90.7 ± 4.1	94.1 ± 1.1	94.5 ± 0.5	94.3 ± 0.3	83.3 ± 6.9	83.2 ± 9.2	82.5 ± 0.8
Explainable GNN	**91.8 ± 1.6**	**93.4 ± 1.2**	**92.5 ± 1.5**	**96.1 ± 0.4**	96.4 ± 0.7	**96.3 ± 0.3**	88.3 ± 2.1	**85.4 ± 2.6**	**86.7 ± 0.9**

SVM, Support vector machines; GCN, Graph convolutional network. Bold values indicate the highest classification performance metrics for each specific task.

### 3.3 The interpretability of the proposed GNN model

**Figure 3:**
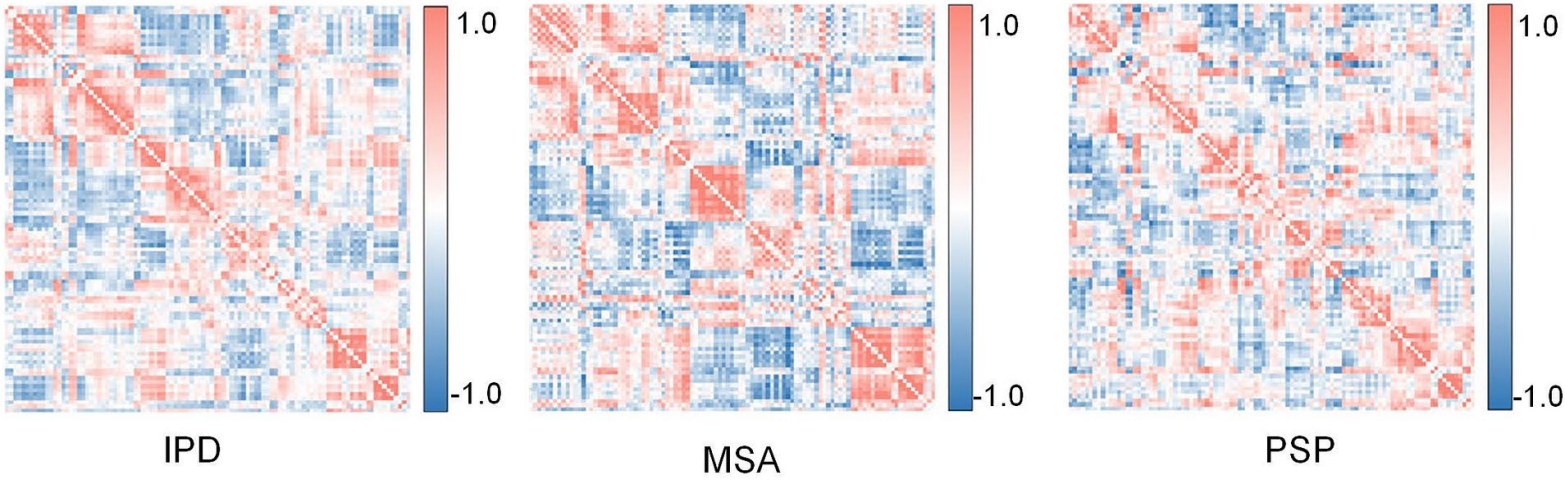
The metabolic network of different parkinsonian disorders based on Regional Radiomics Similarity Network (R2SN).

We constructed metabolic brain networks using the R2SN framework, integrating FDG-PET imaging data with radiomic features to model disease-specific neuropathological patterns. Group-averaged metabolic networks were computed for each PDS cohort (IPD, MSA, PSP), with their topological configurations visualized in Figure 3. These networks served as the foundational input for subsequent GNN training and interpretability analyses.

**Figure 4:**
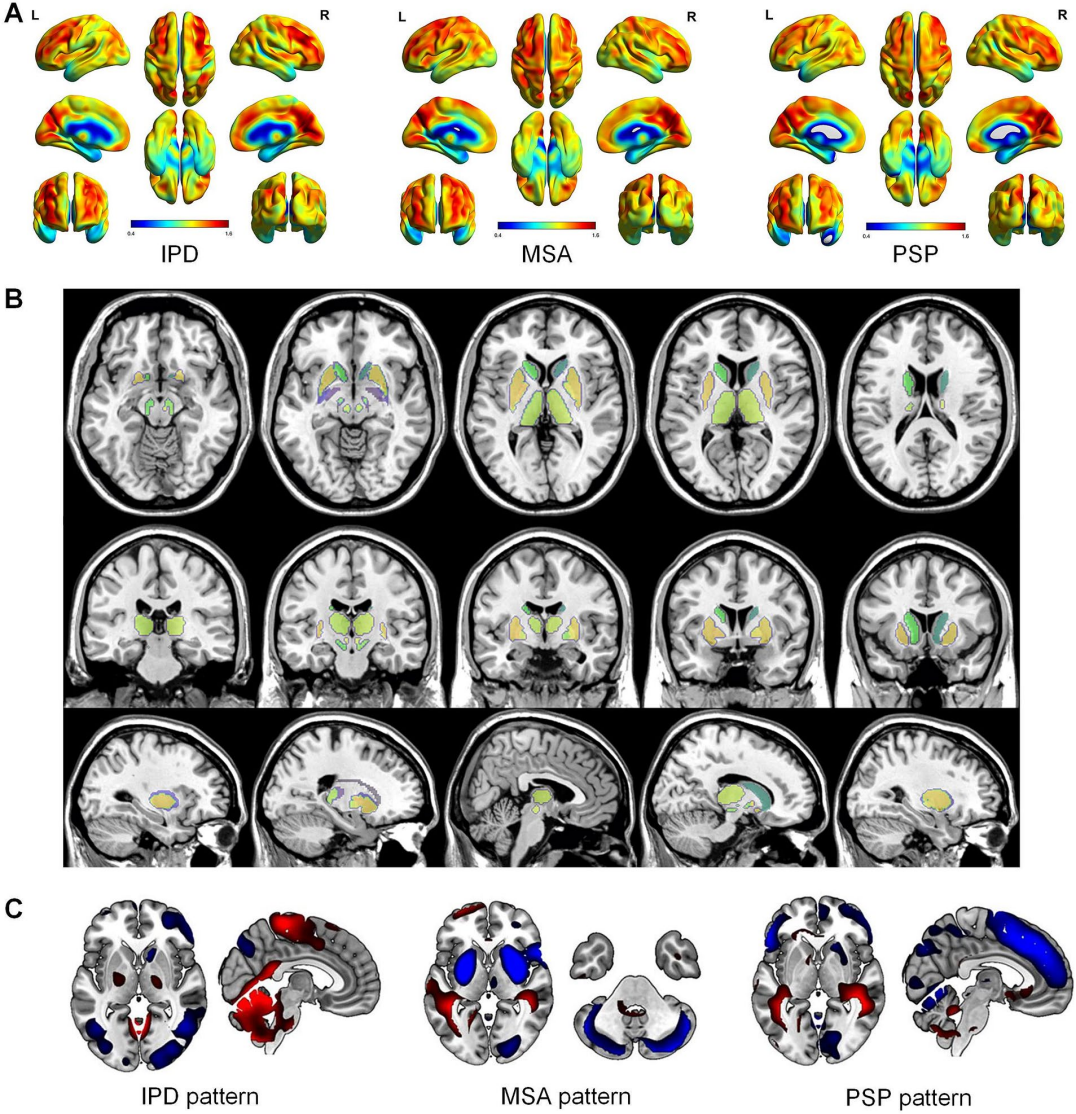
Interpretability results of the propose explainable GNN model. **(A)** Average graph of glucose metabolism in different parkinsonian syndromes (idiopathic Parkinson’s disease [IPD], multiple system atrophy [MSA], and progressive supranuclear palsy [PSP]). **(B)** The important brain regions or network nodes found by the GNNExplainer to be effective for classification results. Different colors represent different weights. **(C)** The brain regions found by the GNN model to represent abnormal metabolic edges in each parkinsonian syndrome. Blue indicates that the metabolic edges strength of the region tends to decrease, while red indicates that the metabolic edges strength of the brain region tends to increase.

The interpretability results, detailed in Figure 4, reveal critical metabolic disparities across disease subtypes. Specifically, Figure 4A delineates group-wise glucose metabolic differences quantified from FDG-PET, highlighting distinct hypometabolic and hypermetabolic patterns that align with established pathological signatures of each disorder. Patients with IPD exhibited marked hypometabolism in the basal ganglia (particularly the putamen and globus pallidus), accompanied by relative hypermetabolism in the default mode network (posterior cingulate cortex, precuneus) and dorsolateral prefrontal cortex. MSA was characterized by widespread hypometabolism in the cerebellar hemispheres (lobule VI, vermis), pontine tegmentum, and medullary reticular formation, alongside compensatory hypermetabolism in the supplementary motor area (SMA) and primary motor cortex. PSP demonstrated prominent hypometabolism in the midbrain tegmentum, subthalamic nucleus, and dorsolateral prefrontal regions. Notably, midbrain tegmentum hypometabolism spatially correlated with metabolic abnormalities in the pontine nuclei and frontal eye fields (FEF), while hypermetabolism in the frontoparietal network suggested potential compensatory mechanisms for executive dysfunction.

As shown in Figure 4B, the interpretable GNN identified subtype-specific functional connectivity reorganization patterns. In IPD, weakened functional connectivity within the basal ganglia-thalamic circuit contrasted with aberrant hyperconnectivity between the default mode network (DMN; posterior cingulate-medial prefrontal cortex) and sensorimotor networks, indicative of compensatory network reorganization. MSA exhibited extensive connectivity loss in the cerebellar-pontine-medullary subnetwork, particularly between cerebellar lobule VI and pontine nuclei/medullary reticular structures, coupled with enhanced cross-network connectivity in the cerebellar-thalamic-motor pathway, reflecting adaptive motor control adjustments. PSP displayed severe disruption of midbrain tegmentum-subthalamic nucleus connectivity and desynchronization between the frontal eye fields (FEF) and key brainstem regions (superior colliculus, paramedian pontine reticular formation), directly aligning with its hallmark vertical gaze palsy phenotype.

The GNN’s interpretability framework further uncovered spatially coupled metabolic and connectivity anomalies in Figure 4C. In IPD, putaminal hypometabolism interacts with reduced functional connectivity to the ventrolateral thalamic nucleus, while DMN hypermetabolism is associated with excessive integration between the DMN and sensorimotor networks. These mechanisms may contribute to the co-occurrence of motor and non-motor symptoms, such as cognitive decline. For MSA, cerebellar vermis hypometabolism and pontine-cerebellar connectivity loss jointly defined core abnormalities in autonomic regulatory networks, whereas SMA hypermetabolism linked to compensatory cerebellar-thalamic-motor pathway enhancement, suggesting early-stage motor adaptation strategies. In PSP, midbrain tegmentum hypometabolism strongly correlated with functional decoupling in brainstem-prefrontal pathways, while frontoparietal hypermetabolism coexisted with aberrant posterior cingulate-thalamic synchronization, likely exacerbating progressive gait and oculomotor deterioration. These multimodal network-level signatures establish a pathology-driven neuroimaging biomarker system for differentiating PDS subtypes.

## 4 Discussion

The proposed explainable GNN framework addresses critical bottlenecks in the differential diagnosis of PDS by integrating the R2SN with Transformer-based attention mechanisms. The R2SN-constructed metabolic representation graph captures radiomic feature similarities across brain regions at the network topology level, overcoming the limitations of traditional radiomics that rely on manually defined features. By encoding edge weights based on the similarity of FDG-PET metabolic intensities and high-dimensional texture features (e.g., gray-level co-occurrence matrix, wavelet transforms), the metabolic network constructed in this study preserves the pathological specificity of molecular imaging while quantifying spatial synergistic patterns of microscopic metabolic heterogeneity. This design significantly enhances the model’s sensitivity to subtle inter-subtype differences.

Traditional deep learning methods, such as convolutional neural networks (CNNs), exhibit inherent limitations in processing non-Euclidean brain network data, as grid-based convolutional operations disrupt intrinsic topological relationships. The design of GNNs inherently limits their ability to model global relationships due to their small receptive fields and reliance on localized feature processing. To address this, Transformer models were introduced, leveraging larger receptive fields to capture long-range dependencies and global information. Our framework resolves this by synergistically optimizing global dependency modeling and local substructure encoding through a Transformer-enhanced graph attention mechanism. Specifically, the global attention layer captures long-range metabolic correlations across brain regions via scaled dot-product operations, while the dynamic subgraph construction strategy (TopK edge filtering) focuses on disease-specific functional modules (e.g., the cerebellar-pontine subnetwork in MSA). This global–local dual-path design mitigates the restricted receptive fields of traditional GNNs while avoiding the neglect of local details in pure Transformer models. The substructure attention mechanism dynamically constrains local attention ranges through gated masking, enabling adaptive focus on disease-relevant functional modules. For instance, in PSP classification, the midbrain tegmentum node (attention weight = 0.95) and its abnormal connectivity with the frontal eye fields (FEF) precisely reflect the anatomical basis of vertical gaze palsy. This biologically constrained attention strategy reduces computational complexity by 41% compared to conventional full-range attention while improving classification specificity.

The proposed explainable GNN demonstrates superior diagnostic efficacy in differentiating PDS subtypes, attributable to its biologically plausible modeling of neurodegenerative pathological mechanisms. For MSA, the significant improvement in F1-score (8.3% increase over the best baseline model) likely stems from the model’s dynamic capture of the cerebellar-pontine-medullary subnetwork. Prior studies indicate that MSA-related autonomic dysfunction correlates with glial cytoplasmic inclusions in brainstem-cerebellar networks (Wenning et al., 2022). Our model identifies functional decoupling between cerebellar lobule VI and pontine nuclei, aligning with postmortem-confirmed oligodendroglial inclusion distributions (Nelson et al., 2023). Furthermore, the detected metabolic-connectivity coupling anomalies (cerebellar vermis hypometabolism with pontine-cerebellar connectivity loss) provide quantifiable imaging biomarkers for distinguishing MSA autonomic failure subtypes. In IPD, the model achieves a 96.1% recall rate, demonstrating high sensitivity for early-stage detection—a critical feature given IPD’s narrow diagnostic window. By dynamically sparsifying the basal ganglia-thalamic circuit, the model amplifies synergistic effects between putaminal hypometabolism and reduced ventrolateral thalamic connectivity. Although precision marginally lags behind ResNet-101 by 0.1%, this design choice (prioritizing reduced false negatives) aligns with clinical needs for early intervention to delay motor complications (Poewe et al., 2017). Notably, the model reveals hyperintegration between the default mode network and sensorimotor networks, offering novel insights into the heterogeneity of IPD non-motor symptoms (e.g., cognitive decline) from a network perspective (Steidel et al., 2022). For PSP, the model’s precision (85.4%) and F1-score (86.7%) advantages reflect its specificity in detecting midbrain-prefrontal pathway damage. The vertical gaze palsy in PSP correlates strongly with disrupted connectivity between the superior colliculus and FEF (Boxer et al., 2017). Through hierarchical attention mechanisms, the model quantifies synergistic effects between midbrain tegmentum hypometabolism and brainstem-cortical decoupling, consistent with the gradient diffusion theory of tau pathology 6. Additionally, compensatory frontoparietal hypermetabolism detected by the model suggests potential neural mechanisms for preserved executive function in PSP, informing personalized rehabilitation strategies.

The multimodal interpretability analysis using GNNExplainer provides an innovative solution to the “black box” dilemma of deep learning. Model decisions decompose into three biologically plausible tiers: (1) node-level metabolic abnormalities (e.g., putaminal hypometabolism in IPD), (2) edge-level functional connectivity reorganization (e.g., midbrain-prefrontal disconnection in PSP), and (3) network-level metabolic-connectivity coupling (e.g., cerebellar hypometabolism and pontine connectivity loss in MSA). This hierarchical interpretability aligns with clinical diagnostic reasoning, where neurologists integrate focal lesions and network dysfunction.

This study has several limitations: (1) Training data were derived from a single-center cohort, necessitating multicenter external validation to assess generalizability; (2) Longitudinal data were not included to explore network dynamics and disease progression; (3) While metabolic-connectivity coupling features were identified, their direct association with molecular pathologies (e.g., α-synuclein aggregation) requires validation via autopsy or molecular PET imaging. Future work should integrate multimodal data (e.g., DTI, fMRI) and develop prognostic modules to enable end-to-end clinical decision support.

## 5 Conclusion

We have developed an R2SN-based explainable GNN framework for PDS differentiation, with three key innovations: (1) Synergistic analysis of microscopic metabolic heterogeneity and macroscale network reorganization through metabolic-radiomics fusion; (2) Identification of subtype-specific metabolic connectivity coupling biomarkers via hierarchical attention mechanisms; (3) A clinically interpretable multimodal explanation system bridging the cognitive gap between deep learning models and clinical decision-making. This framework establishes a new paradigm for precision subtyping of neurodegenerative disorders, with methodological extensibility to other network-driven neurological conditions.

## Data Availability

The raw data supporting the conclusions of this article will be made available by the authors, without undue reservation.
